# Pharyngoesophageal Suturing Technique May Decrease the Incidence of Pharyngocutaneous Fistula following Total Laryngectomy

**DOI:** 10.1155/2015/363640

**Published:** 2015-08-05

**Authors:** Mahmut Deniz, Zafer Ciftci, Erdogan Gultekin

**Affiliations:** Department of Otorhinolaryngology, School of Medicine, Namik Kemal University, 59100 Tekirdag, Turkey

## Abstract

*Objectives*. A pharyngocutaneous fistula (PCF) following total laryngectomy is associated with increased morbidity and severe life threatening complications. We aimed to review our experience with the PCF following total laryngectomy and determine the impact of previously reported risk factors on the development of PCF in our patients. *Methods*. The medical records of 20 patients who had a total laryngectomy operation were retrospectively analyzed. The association between the proposed risk factors and the incidence of the PCF was investigated. *Results*. Comparison of the suture techniques used for the closure of the pharynx (either continuous Cushing type or interrupted) yielded that primary interrupted sutures had a significantly higher incidence of PCF formation (*p* < 0.05). Although it was not statistically significant, diabetes mellitus was also associated with increased PCF formation (*p* > 0.05). No significant difference was observed between the PCF and non-PCF groups in terms of other proposed risk factors (*p* > 0.05).  *Conclusions*. The main risk factor associated with PCF was found to be the type of pharyngeal closure technique. A vertical closure with a Cushing type continuous suture may be more successful than interrupted sutures in preventing a PCF.

## 1. Introduction

In the recent years, the opportunity to preserve the functions of vocalization, swallowing, and natural airway respiration by organ preserving approaches led to a decrease in the number of total laryngectomy operations performed for laryngeal cancer [1]. However, for advanced stage tumors or recurrent disease, despite its significant morbidity and high complication rates, a total laryngectomy operation is usually employed [2]. Following total laryngectomy, a number of complications including, but not limited to, wound infection, swallowing difficulties, compromise of the airway, chyle leak, carotid rupture, and pharyngocutaneous fistula (PCF) may be seen in the early postoperative period [3]. Among these complications, the PCF merits special attention due to its significant negative impact on the recovery process of the patient and relatively high incidence rates ranging from 5 to 65 percent [4]. The PCF was found to be associated with increased hospitalization time, delayed adjuvant postoperative therapy, nutritional deterioration of the patient, and severe life threatening complications [5].

So far, in the literature, many factors were proposed to be significantly associated with the development of the PCF. Advanced primary tumor stage, preoperative radiotherapy, duration of surgery, transfusion requirement, patient comorbidities, prior tracheotomy, low perioperative albumin and hemoglobin, hypothyroidism, presence of tumor beyond resection margins, the type of the suture material, and the type of closure technique were implicated in the development of the PCF [2, 6, 7]. Despite the abundance of the series emphasizing the role of the various risk factors, the findings of these studies are usually inconsistent with the findings of the previous literature and a consensus regarding the identification of significant risk factors could still not be established.

The purpose of this retrospective analysis was to review our single-institute based experience with the PCF following total laryngectomy and determine the impact of previously reported risk factors on the development of PCF in our patients. The implications of our findings were also discussed within the scope of the existing literature.

## 2. Materials and Methods

The medical records of the patients, who underwent total laryngectomy for squamous cell carcinoma of the larynx in a tertiary referral center between 2010 and 2015, were retrospectively reviewed. Data regarding the age, gender, smoking habit, tumor stage, previous radiotherapy or chemotherapy, comorbid conditions including diabetes mellitus (DM), chronic obstructive pulmonary disease (COPD), chronic renal failure (CRF), perioperative hemoglobin, albumin, and thyroid hormone levels, prior tracheotomy, unilateral or bilateral neck dissection, the type of suture material used, and the type of closure technique were collected.

Patients who developed a PCF in the postoperative period (the PCF group) were considered as the study group and they were compared with the remaining patients (the non-PCF group). The main variables of the patients in both groups are listed in Table 1.

**Figure 1:**
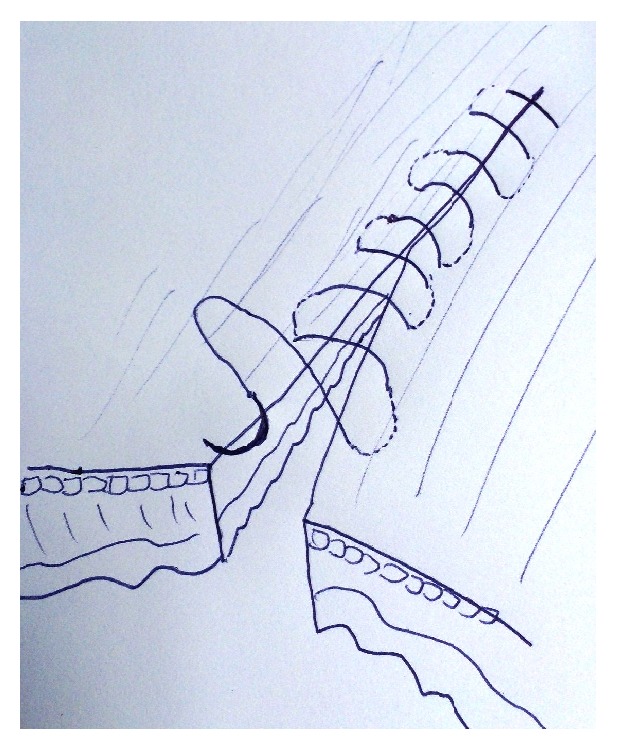
Cushing type continuous suture.

**Figure 2 fig2:**
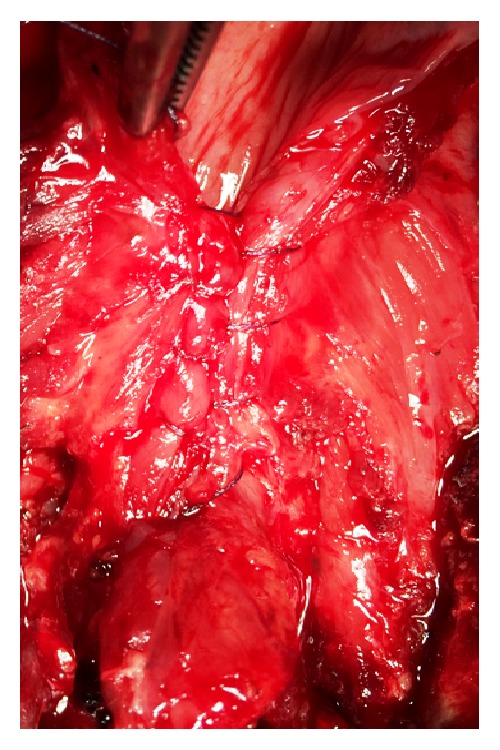
Closure of the pharyngoesophageal segment using a Cushing type continuous suture.

**Figure 3:**
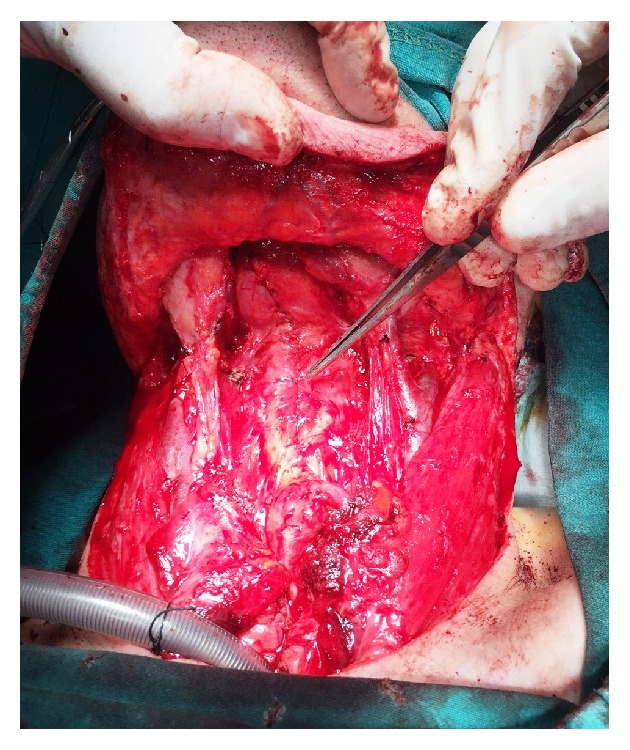
Reconstruction of the neopharynx was accomplished by a vertical closure using a Cushing type continuous suture.

Total laryngectomy and pharyngeal closure were accomplished by the senior surgeons in all patients. Except for the type of suturing technique employed for creating the neopharynx, all patients underwent similar surgical interventions. All mucosal defects were closed by primary sutures and no patients required a flap procedure for the closure of the pharyngoesophageal segment. A 3/0 resorbable suture, “Surgicryl 910, HR-17 round bodied taper point needle 17 mm,” manufactured from Polyglactine 910 was used as the suture material for all patients (SMI AG, Belgium). A vacuum drainage system was kept in place for 48 hours and a nasogastric tube was inserted. All patients received sulbactam/ampicillin 1 g/6 h i.v. after the procedure until 72 h later. For the creation of the neopharynx, either a vertical closure with a Cushing type continuous suture (Figures 1 [Fig fig2]–3) or a T shaped closure with interrupted sutures was preferred.

For statistical analysis, SPSS for Windows, version 17, was used (SPSS Inc., Chicago, IL). The comparison of the qualitative data was conducted using Fisher's exact test. Results were considered as significant at the level where *p* < 0.05.

## 3. Results

The study population included 20 patients (1 female and 19 males) who underwent a total laryngectomy operation for stage 4 laryngeal cancer. The mean age of the patients was 58 years (range 51–68 years). The median follow-up time for all patients was 13.7 months (range 6.2–24.1 months). All patients had a history of smoking (at least 20 cigarettes per day for 20 years).

The PCF was observed in 4 of 20 patients (20%). Of these 4 patients, 1 patient was female and three were male. An additional surgical procedure was required to close the PCF in one patient and the remaining three patients had spontaneous closure of the PCF within one month.

13 patients had their pharyngeal closures with a Cushing type suture and none of them developed PCF in the postoperative period (0%). The pharyngeal closures of the remaining 7 patients were performed using interrupted sutures and, in this group of patients, 4 patients (57.14%) developed a PCF within the first postoperative week (Table 1). A statistically significant difference was observed between the two different suture groups in terms of PCF formation (*p* = 0.007, *p* < 0.05).

Four of 20 patients had a history of DM in the study group. Only one diabetic patient was in the PCF group (25%). In the non-PCF group, 3 patients were diabetic (18.75%). Although the incidence of PCF was slightly higher among the diabetic patients, the difference was not statistically significant (*p* = 0.624, *p* > 0.05).

Serum albumin levels were normal in 17 of 20 patients and 4 patients in this group had a PCF (23.52%). Hypoalbuminemia (serum albumin level <3.2 g/dL) was present in 3 of 20 patients (15%) and none of them had a PCF (0%) (*p* = 0.596, *p* > 0.05). Perioperative serum hemoglobin levels were found to be within normal range in 16 of 20 patients (80%) and 2 patients developed a PCF in this group (12.5%). Although the remaining 4 patients had low hemoglobin levels, no patient in this group had a PCF in the postoperative period (0%). The association between low perioperative hemoglobin and development of a PCF was not statistically significant (*p* = 0.491, *p* > 0.05). Perioperative thyroid hormone levels were normal in all the patients.

15 of 20 patients were found to have a prior tracheotomy. Three of 15 patients (20%) with a prior tracheotomy and 1 of 5 patients (20%) without a prior tracheotomy were found to develop a PCF. The difference between the groups was statistically insignificant (*p* = 0.751, *p* > 0.05).

All patients in the study group had a bilateral neck dissection. None of the patients had a history of either chronic renal failure or preoperative chemotherapy or radiotherapy.

## 4. Discussion

In this research, we retrospectively investigated the impact of previously reported risk factors on the development of a PCF following total laryngectomy. Among these risk factors, although conflicting studies were reported in the literature, the type of suturing technique used for pharyngeal closure was suggested to be a significant risk factor [8, 9]. In this analysis, 57.14% of patients who had their pharyngeal closures with interrupted sutures were found to have a PCF. Strikingly, none of the patients who had a pharyngeal closure with a Cushing type continuous suture developed a PCF. This finding was also consistent with the previous reports emphasizing the high success rates and ease of application of a continuous type suturing technique following anastomosis or repair of the wall of the esophagus [10, 11]. In gastrointestinal system surgery, continuous suturing techniques (Connell and Cushing suture) are widely used for ileal, jejunal, and other colonic anastomoses either for cancer resection or traumatic perforations and successful results were presented in the literature [12, 13]. Pharyngoesophageal junction is the entry point for the gastrointestinal system; therefore, it is reasonable to assume that using Cushing type suture to join the pharyngeal and esophageal segments should be more successful than interrupted sutures in the prevention of a PCF following total laryngectomy.

The presence of a comorbid medical condition including DM, COPD, and CRF was proposed to be a significant risk factor for the development of the PCF [14]. However, such an association could not be demonstrated by others [15]. The PCF incidence in patients with a history of DM, although statistically insignificant, was higher in our study. On the contrary, none of the patients who developed a PCF had a history of either COPD or CRF. Larger series should be conducted to establish such an association.

The impact of low perioperative hemoglobin and albumin levels on the PCF incidence was also extensively reviewed in the literature [16]. However, in our study, perioperative anemia and hypoalbuminemia were not significantly associated with the PCF formation (*p* > 0.05).

Addition of neck dissection to total laryngectomy is another suggested contributing factor for the development of a PCF [17, 18]. In one study, the incidence of the PCF was reported to be increased from 11.3% to 17.5% when neck dissection is combined with total laryngectomy [19]. In our study, all patients in the PCF and non-PCF groups had a bilateral neck dissection and the impact of neck dissection on the incidence of the PCF could not be investigated.

Another factor that was proposed to increase the incidence of a PCF was having a prior chemotherapy or radiotherapy. It was suggested that preoperative chemotherapy or radiotherapy was also associated with longer hospital stays and the necessity for a second surgical operation for the closure of PCF was higher in preoperatively irradiated patients [20–22]. Other authors, however, could not find such an association [23, 24]. In our study, the impact of preoperative chemo- or radiotherapy on the PCF incidence could not be evaluated because none of the patients in our study group had a preoperative treatment.

A preoperative tracheotomy was found to be responsible for the increased rates of PCF following laryngectomy [25, 26]. In the present study, we could not reveal such an association because no statistically significant difference was present between the two different groups of patients in terms of PCF formation (*p* > 0.05). We are of the opinion that larger series should be conducted to further analyze such an association.

## 5. Conclusion

Pharyngocutaneous fistula is one of the most common postoperative complications among the patients who underwent total laryngectomy. The development of the PCF significantly increases the length of the hospital stay and the incidence of severe life threatening complications in this group of patients. In our research, the main factor associated with the occurrence of this complication was found to be the type of suturing technique used for pharyngeal closure. A vertical closure with a Cushing type continuous suture may be more successful than a T shaped closure with interrupted sutures in decreasing the incidence of a PCF following total laryngectomy.

## Figures and Tables

**Table 1:** The main variables of the patients in both groups.

Risk factors	PCF group	Non-PCF group	Total
Interrupted suture Continuous suture	4	3	7
	0	13	13

DM^*∗*^ (+) DM (−)	1	3	4
	3	13	16

Hypoalbuminemia (+) Hypoalbuminemia (−)	0	3	3
	3	11	17

Preop. tracheotomy (+) Preop. tracheotomy (−)	3	12	15
	1	4	5

Neck dissection (+) Neck dissection (−)	4	16	20
	0	0	0

Preop. radiotherapy (+) Preop. radiotherapy (−)	0	0	0
	4	16	20

COPD^*∗∗*^ (+) COPD (−)	0	2	2
	4	14	18

Anemia (+) Anemia (−)	0	3	3
	4	13	17

CRF^*∗∗∗*^ (+) CRF (−)	0	0	0
	4	16	20

Stage 4 cancer (+) Stage 4 cancer (−)	4	16	20
	0	0	0

Hypothyroidism (+) Hypothyroidism (−)	0	0	0
	4	16	20

^*∗*^Diabetes mellitus: DM, ^*∗∗*^chronic obstructive pulmonary disease: COPD, and ^*∗∗∗*^chronic renal failure: CRF.
